# The First District Dental Society of the State of New York

**Published:** 1885-11

**Authors:** 


### THE FIRST DISTRICT DENTAL SOCIETY OF THE STATE OF NEW YORK.

As an item of "Current News," we publish the programme issued by the First District Dental Society for the current society year. It will well repay the careful study of the members of other professional societies, for in it may be found a key to the success of one of the best of our professional organizations.

The first thing to be noted is the fact that the whole programme for the year is made out and published in one circular. Thus all the work of the society is brought to the notice of the members and the profession, and a chance is afforded to see what is accomplished as a whole. An opportunity is also given for members to prepare for a coming meeting some time in advance, and to make notes of cases in practice and of instances from their reading for presentation at the proper time. Something illustrative of a subject that is to be discussed, perhaps months ahead, may be encountered and entered in the appointment book or diary under the proper date, and thus its presentation will be assured. Essayists have abundant time for the study of their subject, and better papers will be secured. Visitors, and those who can attend only occasionally, will be enabled to select the time for their attendance, and to be present when subjects in which they are particularly interested will be considered. The heaviest work of the committees having been thoroughly done in advance, they will be enabled to give more attention to details, and will be spared the great annoyance of running around at the last moment to secure essayists and speakers, who can at best, under such circumstances, be but illy prepared for their duties. In short, the advantages of laying out the work and publishing the programme early are manifold, and must certainly result in successful meetings.

A glance at the list of essayists gives another insight into the reason for the success of the society. The speakers are chosen from among the foremost men of the profession. They are not selected from the city of New York alone, but wherever a man is found who has made unusual progress in any special department, his knowledge is placed at the disposal of the members. The whole professional field is ransacked to find those who have something worthy to offer to the society. Every facility for the illustration of the subject is afforded, and when the discussion comes the essayist is sure that it will be intelligently debated.

Some idea of the strength of the society will be gained, when it is known that at the October meeting more than one hundred and fifty members and visitors were present.

We hope that this society will continue to make the meetings better and better, from year to year, and that they will give their proceedings the widest publicity. Their sayings and doings belong to the whole profession. As representative men they have no right, either professional or moral, to keep back their rich stores of experience from any dentist who desires instruction, and we are sure that they do not desire to deny to any humble member of the profession the light which he sorely needs for his illumination.

